# Human Neural Stem Cells Differentiate and Promote Locomotor Recovery in an Early Chronic Spinal coRd Injury NOD-*scid* Mouse Model

**DOI:** 10.1371/journal.pone.0012272

**Published:** 2010-08-18

**Authors:** Desirée L. Salazar, Nobuko Uchida, Frank P. T. Hamers, Brian J. Cummings, Aileen J. Anderson

**Affiliations:** 1 Department of Anatomy and Neurobiology, University of California Irvine, Irvine, California, United States of America; 2 Sue and Bill Gross Stem Cell Research Center, University of California Irvine, Irvine, California, United States of America; 3 Reeve-Irvine Research Center, University of California Irvine, Irvine, California, United States of America; 4 StemCells, Inc., Palo Alto, California, United States of America; 5 Rehabilitation Hospital Tolbrug, 's Hertogenbosch, The Netherlands; 6 Department of Physical Medicine and Rehabilitation, University of California Irvine, Irvine, California United States of America; University of Milan-Bicocca, Italy

## Abstract

**Background:**

Traumatic spinal cord injury (SCI) results in partial or complete paralysis and is characterized by a loss of neurons and oligodendrocytes, axonal injury, and demyelination/dysmyelination of spared axons. Approximately 1,250,000 individuals have chronic SCI in the U.S.; therefore treatment in the chronic stages is highly clinically relevant. Human neural stem cells (hCNS-SCns) were prospectively isolated based on fluorescence-activated cell sorting for a CD133^+^ and CD24^−/lo^ population from fetal brain, grown as neurospheres, and lineage restricted to generate neurons, oligodendrocytes and astrocytes. hCNS-SCns have recently been transplanted sub-acutely following spinal cord injury and found to promote improved locomotor recovery. We tested the ability of hCNS-SCns transplanted 30 days post SCI to survive, differentiate, migrate, and promote improved locomotor recovery.

**Methods and Findings:**

hCNS-SCns were transplanted into immunodeficient NOD-*scid* mice 30 days post spinal cord contusion injury. hCNS-SCns transplanted mice demonstrated significantly improved locomotor recovery compared to vehicle controls using open field locomotor testing and CatWalk gait analysis. Transplanted hCNS-SCns exhibited long-term engraftment, migration, limited proliferation, and differentiation predominantly to oligodendrocytes and neurons. Astrocytic differentiation was rare and mice did not exhibit mechanical allodynia. Furthermore, differentiated hCNS-SCns integrated with the host as demonstrated by co-localization of human cytoplasm with discrete staining for the paranodal marker contactin-associated protein.

**Conclusions:**

The results suggest that hCNS-SCns are capable of surviving, differentiating, and promoting improved locomotor recovery when transplanted into an early chronic injury microenvironment. These data suggest that hCNS-SCns transplantation has efficacy in an early chronic SCI setting and thus expands the “window of opportunity” for intervention.

## Introduction

Traumatic spinal cord injury (SCI) results in partial or complete paralysis along with sensory loss below the level ofinjury. The pathology of SCI is characterized by the loss of neurons and oligodendrocytes, axonal injury, and demyelination/dysmyelination of spared axons. Therapeutic transplantation of stem cell populations may promote functional recovery by providing trophic support, modifying the host environment to create a permissive environment for endogenous regeneration/repair, or by replacing neurons and/or oligodendrocytes [1], [2].

SCI therapies can target acute, sub-acute, or chronic time points post-injury. The continuum from acute to chronic injury both in animal models and clinically is defined by the transition from a dynamic to a relatively stable injury environment, and when behavioral recovery reaches a plateau [3], [4], [5]. In rodent contusion injury models these criteria are met beginning at approximately 30 days post-injury (dpi) [3], [4], [5]. There are over 1,275,000 individuals living with chronic SCI in the U.S. alone (Christopher & Dana Reeve Foundation Paralysis Resource Center); thus, a chronic transplantation model is highly clinically relevant.

Several studies have investigated chronic SCI models using whole tissue grafts and peripheral nervous system (PNS) cells. Transplantation of fetal spinal tissue, fetal brain cortex, olfactory ensheathing cells (OECs), peripheral nerve grafts, and Schwann cells after SCI have all been shown to improve locomotor recovery [6], [7], [8], [9], [10], [11], suggesting that the chronic post-injury period may be a feasible target for repair.

In contrast, the few studies that have compared sub-acute and chronic transplantation of CNS cell populations such as human oligodendrocyte progenitor cells (OPCs) and mouse neural stem cells (NSCs) in chronic SCI models have not reported improved locomotor recovery [12], [13]. Human OPCs transplanted 7 dpi survived and promoted locomotor recovery; however, at 10 months post-injury, OPCs survived but failed to improve locomotor recovery [12]. Mouse NSCs transplanted 2 weeks post-SCI survived and improved locomotor recovery; however, at 2 months post-SCI, NSCs neither survived nor improved locomotor recovery [13].

Thus, while whole tissue grafts and PNS cells have shown some capacity for chronic stage repair (≥4 weeks post-SCI in rodents), CNS cell populations have thus far failed in the chronic setting. These studies suggest that the mechanism of cell transplant-mediated repair, the properties of specific cell transplant populations, and/or the microenvironment of the injured niche during the acute, sub-acute, and chronic periods may influence the potential to impact recovery post-SCI. Defining the potential window for successful engraftment and recovery in animal models with specific cell populations, particularly CNS populations, is therefore a critical step to developing therapeutics for chronic injuries.

We have previously reported that NOD-*scid* mice, which are constitutively immunodeficient, lacking a normal T-cell, B-cell, and complement response, exhibit similar SCI pathology and cellular innate immune response to other mouse strains (C57Bl/6 and BUB/BnJ) [14]. Accordingly, NOD-*scid* mice provide an excellent experimental model to investigate the potential of transplanted human cell populations to engraft and promote histological and locomotor recovery following SCI without a xenograft rejection response [15]. Furthermore, NOD-*scid* mice have been used as a host for induced pluripotent cells in the CNS as an assay for tumor formation and NSC transplantation studies [16], [17]. Hence, stem cell transplantation in the CNS using the NOD-*scid* model can provide tumorigenicity information. We have previously reported on the sub-acute transplantation of human neural stem cells (hCNS-SCns), which are lineage restricted to generate neurons, oligodendrocytes, and astrocytes, into a NOD-*scid* SCI model. hCNS-SCns are prospectively isolated based on fluorescence-activated cell sorting (FACS) for a CD133^+^ and CD24^−/lo^ population from fetal brain and grown as neurospheres [18].

hCNS-SCns transplanted sub-acutely 9 dpi in immunodeficient NOD-*scid* mice successfully engrafted and improved long-term locomotor recovery compared to vehicle and human fibroblast (hFbs) control groups [19], [20]. Notably, recovery was abolished following selective ablation of hCNS-SCns using diphtheria toxin, demonstrating that survival of hCNS-SCns was required to sustain locomotor recovery [19]. The majority of hCNS-SCns exhibited differentiation to oligodendrocytes, and a smaller percentage differentiated into neurons, but few exhibited evidence of astrocytic fate [19]. This is in contrast to many studies that have demonstrated predominant astroglial fate or differentiation failure following acute or sub-acute NSCs transplantation, [21], [22], [23], [24], [25], [26], [27]. Furthermore, immuno-electron microscopy in the sub-acute study revealed that human cells remyelinated mouse host axons and formed putative synapses with mouse neurons, suggesting that transplanted hCNS-SCns had stably integrated within the functional cytoarchitecture of the host CNS [19].

In the present study, we tested whether hCNS-SCns transplanted into immunodeficient NOD-*scid* mice at an early chronic time point (30 dpi), survived, differentiated, and promoted locomotor recovery. We also investigated whether the animals experienced mechanical allodynia, and stereologically quantified the number of engrafted cells, lesion volume, spared tissue volume, and glial scar area. Our results reveal that hCNS-SCns have the ability to survive, migrate, differentiate, and promote improved locomotor recovery when transplanted in the early chronic SCI microenvironment.

## Methods

### Ethics statement

All animal housing conditions, surgical procedures, and postoperative care was approved by and conducted according to the Institutional Animal Care and Use Committee (IACUC) guidelines at the University of California, Irvine. The UC Irvine Human Stem Cell Research Oversight Committee (UCI hSCRO) approved the use of human stem cells in this study.

### Group design

Female mice were used in this study to avoid bladder complications and urolithiasis that frequently occur in male mice following SCI [14], [28]. Animal surgeries were done in parallel over multiple days. Prior to surgery, NOD-*scid* mice (n = 48) were tested by the Basso Mouse Scale (BMS) to ensure normal locomotor function. One animal was excluded at this time because of abnormal gait. During the initial SCI surgery, one animal died and three additional animals were excluded because of surgical error. Pre-hoc criteria were established to exclude animals if any of the following conditions occurred: cord bruised or tearing of dura during laminectomy (n = 1); visual slippage of the mouse from the vertebral stabilization clamps (n = 0); abnormal time versus force curve indicating a bone hit or clamp slip (n = 1); or unilateral bruising of the cord (n = 1). Mice (n = 44) received BMS testing 2 dpi and weekly thereafter and were randomized into 3 balanced groups, to be transplanted with either vehicle control, hFbs, or hCNS-SCns, based upon their BMS scores at 28 dpi. To minimize the effect of variations in spinal cord damage, seven animals were excluded prior to transplantation because their BMS scores at 28 dpi were more than two standard deviations from the mean score. Thirty-seven mice were transplanted 30 dpi. Slightly more animals were included in the hCNS-SCns group for purposes of histological analysis; vehicle control (n = 11), hFbs (n = 11), and hCNS-SCns (n = 15). Following transplantation, four animals died prior to sacrifice, one from the vehicle group, two from the hFbs group, and one from the hCNS-SCns group. The final animal numbers per group were, vehicle (n = 10), hFbs (n = 9), and hCNS-SCns (n = 14).

### Spinal Cord Injury

Female NOD-*scid* mice (transferred from StemCells Inc, the Jackson Laboratory, stock # 001303), 8–10 weeks of age were anesthetized with tribromoethanol (312.5 mg/kg i.p. bolus) and vertebral T9 exposed by laminectomy as previously described [29]. Mice received a 50-kDyne-contusion injury using the IH device (Precision Systems and Instrumentation LLC). After surgery, mice recovered overnight in cages with Alpha-Dri bedding (Newco Distributors Inc) placed on water-jacketed heating pads at 37°C. Mice were maintained on twice daily manual bladder expression for 2 weeks, followed by once daily manual bladder expression for the remaining survival period. Post-operative care included buprenorphine twice a day for 2 days, lactated ringers once a day for 4 days, and antibiotic daily for the duration of the study. All animals were maintained on rotating schedule of antibiotics; Baytril, Amoxicillin, and Cipro were each given for 2 weeks, and then rotated to the next antibiotic.

### Transplantation surgery

Isolation and culture of hCNS-SCns from fetal brain tissue (gestational 16–20 weeks) have been previously described [18], [19], [30]. Briefly, FACS-sorted single cell suspensions were cultured in neurosphere initiation media consisting of Ex-Vivo 15 media with N2 supplement, FGF, EGF, LIF, neural survival factor-1, and NAC. Cells were propagated as neurospheres, fed weekly, and passaged every 2–3 weeks [18], [19], [30]. Human fibroblasts derived from fetal liver were grown to confluence in Iscove's modified Dulbecco's medium/10% FBS, dissociated with trypsin, washed and concentrated to 50,000 cells per µl. Thirty days post-injury, mice were anesthetized with tribromoethanol (312.5 mg/kg i.p. bolus), the laminectomy site re-exposed and hCNS-SCns or hFbs were transplanted. Four injections of 250 nl each, bilateral from the midline, both rostral and caudal to the injury epicenter were performed using a beveled glass (0.53 mm I.D, 1.14 mm O.D. Drummond Scientific Co.) micropipette (75–80 µm ID, 100–115 µm, 30° bevel Sutter Instrument Co.) and NanoInjector 2000 system (World Precision Instruments) delivering a total of 1 µl of hCNS-SCns at 75,000-cells/µl, or hFbs at 50,000-cells/µl in an injection buffer consisting of 50% Hanks' balanced salt solution and 50% X-vivo medium. Vehicle control mice received injection buffer alone. Mice received post-operative treatment as described above.

### BMS

Open-field locomotor recovery was assessed using the BMS locomotor rating scale prior to injury, 2 days after injury, weekly for 4 weeks until transplantation, then weekly following transplantation until sacrifice. Briefly, mice were observed in the open-field for 4 minutes each by two individuals blinded to the experimental groups [31], [32]. Motor function of the hind limbs was rated, recorded, and converted to a score according to the published scale.

### CatWalk Analysis

CatWalk video was recorded at 16 weeks post-transplantation. Individuals blinded to experimental groups analyzed video using CatWalk software version 6.13 for Windows [33].

### Mechanical Allodynia

Von Frey hair testing of hind limbs was done at 15 weeks post-transplantation. Animals were tested for a withdrawal response to successively higher force filaments (Touch-Test Sensory Evaluators, North Coast Medical). The lowest filament from which an animal withdrew from was the threshold assigned for that animal [34]. After a positive response was elicited, the previous filament was tested to confirm a lack of response and the next filament was tested to confirm a positive response.

### Histological Assessment

Sixteen weeks post-transplantation, mice were anesthetized using pentobarbital (100 mg/kg) and transcardially perfused with 15 mls of PBS followed by 100 mls of 4% phosphate-buffered paraformaldehyde. A T6-T12 segment of the spinal cord was dissected based on counting the dorsal spinal roots for all mice to obtain an anatomically consistent region of the spinal cord for stereological analysis. Spinal roots were identified by visualization of the features of C6, C7 and C8 root brachial plexus and exposure of the distal thoracic roots. Dissected T6-T12 spinal cord segments were post-fixed in a 20% sucrose/4% paraformaldehyde solution for cryoprotection overnight at 4°C. Following cryoprotection, spinal cords were frozen in isopentane at −65°C, and serial sliding microtome sections were collected free floating for immunocytochemical staining. Parasagittal sections (n = 6 per group) were collected at 30 µm for human cytoplasm and paranodal protein (CASPR) double immunofluorescence and stereological analysis of hCNS-SCns and hFbs survival and migration, lesion volume, spared tissue volume, and glial scar area. Coronal sections (n = 3 per group) were collected at 50 µm for fate analysis. Immunocytochemistry was conducted as previously described [35] sampling throughout the T6–T12 segment of the spinal cord using anti-human cytoplasm marker (SC121-StemCells, Inc 1∶4000), anti-GFAP marker (GFAP-Dako 1∶60,000), anti-human GFAP marker (SC123-StemCells,Inc 1∶3000) and visualized using diaminobenzidine (DAB; Vector Laboratories). For fluorescent labeling we utilized anti-human cytoplasm marker (SC121-StemCells, Inc 1∶4000), anti-human nuclei marker (SC101- StemCells, Inc 1∶100), anti-human nuclei marker (Millipore 1∶200), anti-APC-CC1 marker (Calbiochem 1∶4000), anti-human Olig2 (R&D Systems 1∶100), anti-ß-tubulin-III (Convance 1∶5000), anti-GFAP (Dako 1∶30,000), anti-Nestin (Covance 1∶1000), anti-Ki67 (Novocastra 1∶1500), and anti-CASPR (Abcam 1∶500). Secondary antibodies in double-labeling experiments were Alexa Fluor 488 and 555 (Molecular Probes 1∶500). Fluorescent sections were mounted with Vectashield mounting medium for fluorescence with DAPI (Vector Laboratories).

### Stereological Quantification

Stereology was conducted using an Olympus BX51 microscope with a motorized stage and StereoInvestigator software (MBF Biosciences, version 7.00.3). Survival of human cells, hCNS-SCns (n = 6) and hFbs (n = 5), was quantified by SC121 immunolabeling and methyl green nuclear counterstain using the optical fractionator probe and a 100× oil-immersion 1.30 numerical aperture objective. Human cells were quantified in the injury epicenter, spared tissue and sequential 1 mm segments rostral and caudal to the injury site. Systematic random sampling of the tissue was performed according to stereological principles. Starting sections were chosen at random and every sixth section thereafter was analyzed. Sampling parameters (grid and counting frame size) were empirically determined to achieve low coefficients of error (CE) for each measure. CE values are summarized in Table 1. Lesion volume, spared tissue volume, and glial scar area were quantified using GFAP staining and the Cavalieri probe at 4× in all three groups using parasagittal sections, vehicle (n = 5), hFbs (n = 5), and hCNS-SCns (n = 6). Lesion volume was quantified as the area at the injury epicenter that was devoid of GFAP staining using a 100 µm grid. Spared tissue volume was quantified in 500 µm segments rostral and caudal of the injury epicenter using a 100 µm grid. Glial scar area was quantified by measuring the area of dense GFAP staining near the injury epicenter and excluding the lesion as defined by absence of GFAP staining using a 150 µm grid.

**Table 1:** Intra-animal variability in stereological analyses.

	*Parameter of Analysis*			
	hCNS-SCns/hFbs Number	Lesion Volume	Spared Tissue	GFAP Scar
Mean Coefficient of Error (CE)	<0.0580**/**<1 per region	0.0466	0.0295	0.0334

### Quantification of human cell differentiation

hCNS-SCns differentiation was examined by double fluorescent labeling. Confocal imaging of fluorescent stained sections was conducted using a Zeiss LSM 510 Meta confocal system and Zeiss LSM 510 software (Version 4.0 SP2) with multi-track scanning. Three animals for each protein marker were utilized. For quantification of each protein marker, starting sections were chosen at random and every twelfth coronal section thereafter was stained. From the 1 in 12 sampling of sections stained, the ten consecutive sections that contained the most human cells were utilized for fate analysis. In each of the ten consecutive sections, ten fields were imaged using a 63× objective and 2× digital zoom. The number of human cells, and double-labeled cells were counted for each series using ImageJ Version 10.2 software with a cell counter plug-in. The number of double-labeled cells is expressed as a percentage of the total number of human cells counted in each individual series. For each protein marker analyzed, the values from three animals are averaged together to get the final percentage.

### Statistical Analysis

All means are expressed ± the standard error of the mean. For BMS, repeated measures ANOVA was used to compare scores of vehicle, hFbs, and hCNS-SCns transplanted animals. For BMS, linear single degree of freedom contrast statistics and a Bonferroni/Dunn post-hoc analysis was utilized to determine significance at the termination of the study (week 16). Chi-square analysis was utilized to assess the observed frequency of animals recovering coordination in the open field with a Fisher's exact test. CatWalk gait analysis of swing speed was analyzed with a one-way ANOVA and Fisher's PLSD post-hoc test. A one-way ANOVA was used to assess von Frey, lesion volume, spared tissue volume, and glial scar area for differences between the three groups. StatView (version 5.0.1) and Prism (version 5.0a) were used for statistical analysis; significance was defined as p≤0.05.

## Results

### hCNS-SCns promote locomotor recovery

**Figure 1:**
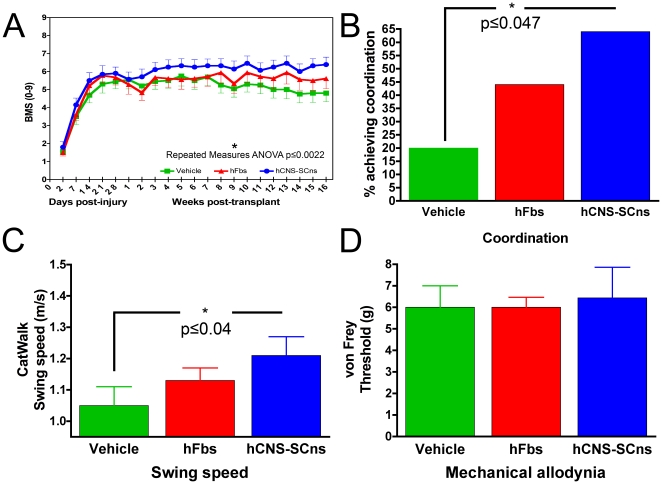
hCNS-SCns promote improved locomotor recovery on multiple tests. (**A**) BMS locomotor performance is significantly improved in hCNS-SCns treated animals compared to vehicle controls (repeated measures ANOVA (p≤0.0022). A Bonferroni/Dunn post-hoc analysis at week 16 revealed a significant difference between hCNS-SCns and vehicle control (p≤0.02). There were no significant differences between hFbs and either hCNS-SCns or vehicle. (**B**) Recovery of coordination was significantly increased in hCNS-SCns treated animals compared to vehicle controls using chi square analysis for observed frequency (p≤0.047, Fisher's Exact Test). No statistically significant differences were found comparing hFbs with vehicle or hCNS-SCns transplanted animals. Error bars are not plotted as these bars represent the absolute percentage of animals reaching criteria. (**C**) CatWalk gait analysis showed that hCNS-SCns treated animals exhibited significantly increased swing speed compared to vehicle treated animals (p≤0.04, ANOVA, Fisher's PLSD). (**D**) von Frey analysis of mechanical allodynia showed no significant differences between any of the groups (p>0.05 ANOVA).

To investigate whether hCNS-SCns can contribute to long-term locomotor recovery in an early chronic SCI model mice received grafts of hCNS-SCns, hFbs, or a vehicle control injection 30 days post contusion injury. All mice were pre-tested for baseline locomotor performance using the BMS prior to SCI and any animals with locomotor deficits were excluded prior to injury (Fig. 1A). After SCI, mice were tested at 2, 7, 14, 21, and 28 dpi, and then weekly following transplantation until sacrifice. Prior to transplantation, mice were randomized into 3 groups based upon BMS scores at 28 dpi, so that each group started with a similar score as described under methods. hCNS-SCns transplanted mice exhibited significantly improved locomotor recovery compared to vehicle control mice; repeated measures ANOVA p≤0.0022 (F = 1.952), Bonferroni/Dunn posthoc analysis at week 16 p≤0.02. There were no significant differences in repeated measures ANOVA between hFbs and either the hCNS-SCns or vehicle control groups. BMS scores were further analyzed using a test of the interaction of the groups across time following transplantation, which was significant (p = 0.002), indicating that the group means changed differently over time. To further characterize what the changes across time were, a linear contrast of the interaction term was determined and was found to be highly significant (p<0.0001), indicating that the groups made linear changes across time with different slopes. Finally, the slope of each group was investigated individually. Based on adjustments for multiple testing, the p-value required to reach significance in this analysis was p≤0.0167. While the vehicle group exhibited a non-significant trend for a linear decrease in means over time (p = 0.023) and the hFbs group exhibited no linear change in means over time (p = 0.115), the hCNS-SCns exhibited a significant linear increase in means over time (p = 0.001) suggesting that only hCNS-SCns treated animals had significantly improved recovery over the 16 weeks following transplantation. At 16 weeks post-transplant (termination of the study) the average BMS scores per group were: hCNS-SCns (n = 14) 6.4±0.4; hFbs (n = 9) 5.6±0.6; and vehicle treated animals (n = 10) 4.8±0.5. These scores correspond to improvement in the frequency of coordinated steps between fore and hind limbs for hCNS-SCns vs. vehicle groups.

Because BMS scores showed differences in the recovery of coordination between groups, we used chi square frequency analysis to compare the number of animals in each group that had regained at least occasional coordination between fore and hind limbs on open-field behavior testing. Data is expressed as the percentage of animals that exhibited coordination between fore and hind limbs out of the total number of animals per group (Fig. 1B). 64% of hCNS-SCns mice recovered coordination compared to 44% for hFbs mice and 20% for vehicle control mice. Recovery of coordination was significantly increased in hCNS-SCns transplanted animals compared to vehicle controls using chi square analysis for observed frequency (p = 0.047, Fisher's Exact Test). No statistically significant differences were found comparing hFbs with either vehicle or hCNS-SCns transplanted mice.

As a supplemental quantitative measure of locomotor recovery, CatWalk gait analysis was performed in a subset of animals at 16 weeks post-transplant prior to sacrifice. CatWalk gait analysis showed that hCNS-SCns treated animals (n = 10) had significantly improved swing speed 1.21 m/s±0.06 compared to vehicle controls (n = 8) 1.05 m/s±0.06 (p<0.04 ANOVA, Fisher's PLSD) (Fig. 1C). However, hFbs treated mice (n = 8) 1.13 m/s±0.04 did not exhibit significant differences from either vehicle control or hCNS-SCns treated mice. Taken together, these data suggest that hCNS-SCns were able to promote improved behavioral recovery following early chronic transplantation.

### hCNS-SCns do not contribute to allodynia

Recent studies have suggested that predominant astrocytic differentiation of transplanted NSCs is associated with the development of allodynia [21], [36], defined as increased sensitivity to stimuli that are normally not noxious, whether improved locomotor recovery was observed or not. In one study [36], allodynia was correlated to the number of NSCs that differentiated into astrocytes. We assessed hind limb mechanical allodynia 15 weeks post-transplant to determine the lowest threshold force from which animals withdrew their paw. Vehicle treated animals exhibited a threshold of 6.0 g±1.0 (n = 9), hFbs 6.0 g±0.5 (n = 9), and hCNS-SCns treated animals 6.4 g±1.4 (n = 10) (Fig. 1D). None of the groups were significantly different from one another by one-way ANOVA (p≥0.94), suggesting that hCNS-SCns treated animals did not have an altered threshold of response to mechanical allodynia as a result of hCNS-SCns transplantation.

### hCNS-SCns survive, engraft, and migrate within the injured spinal cord

**Figure 2:**
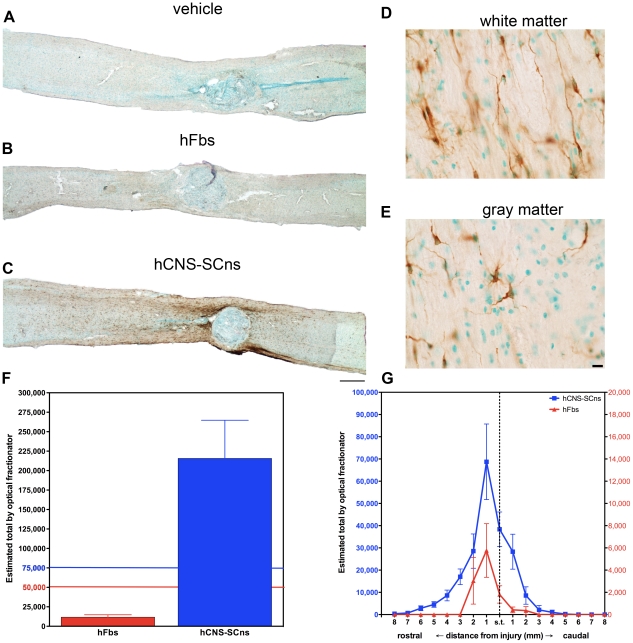
Transplanted hFbs survive and hCNS-SCns survive and migrate extensively. Histology of spinal cords stained for human cytoplasm (SC121, brown and methyl green nuclear counterstain, blue) 16 weeks post-transplantation (**A**) reveal no human cells in vehicle treated animals, (**B**) few human cells in hFbs treated animals, and (**C**) many human cells surviving and migrating the length of the cord in hCNS-SCns treated animals. hCNS-SCns exhibit distinct morphologies in white vs. gray matter. (**D**) In white matter hCNS-SCns exhibit oligodendrocyte-like morphology. (**E**) In gray matter hCNS-SCns exhibit neuronal morphology Scale bar on left: 1000 µm. Scale bar on right: 50 µm. (**F**) 50,000 hFbs were initially transplanted (red line) and 11,701±3070 remain at the termination of the study. 75,000 hCNS-SCns were initially transplanted (blue line) and 215,711±48,978 were present at the termination of the study as estimated using unbiased stereological quantification. (**G**) Unbiased stereological quantification was used to assess the migration of transplanted cells in 1 mm blocks extending 8 mm rostral and caudal from the injury site including the spared tissue (s.t., vertical dashed line) surrounding the lesion. hCNS-SCns migrated up to 8 mm rostral and 5 mm caudal (left y-axis, blue). hFbs migrated 2 mm rostral and 2 mm caudal (right y-axis, red).

To investigate the survival and migration of transplanted cells, mice were sacrificed 16 weeks post-transplantation and engrafted human cells detected using an antibody to human cytoplasm (SC121) (Fig. 2A–E). All transplanted animals exhibited successful human cell engraftment. There were no human cells present in vehicle treated animals and relatively few engrafted hFbs in hFbs transplanted animals, but numerous engrafted hCNS-SCns (Fig. 2A–C). hCNS-SCns were found in both gray and white matter, and the cells were morphologically distinct across these regions. Cells found in the white matter had elongated oligodendrocyte-like morphology (Fig. 2D), while those in the gray matter exhibited neuronal morphologies (Fig. 2E). Engrafted cells were distributed throughout gray and white matter of the cord with no evidence of abnormal morphology or any mass formation indicative of tumorigenesis.

The number of surviving cells was quantified stereologically using the optical fractionator probe. The initial transplant contained 75,000 hCNS-SCns per animal; stereological estimates of hCNS-SCns (n = 6) revealed an average of 215,711±48,978 hCNS-SCns present 16 weeks following transplantation (Fig. 2F). These values represent an approximately 3-fold increase in the initial cell population transplanted. The initial hFbs transplant contained 50,000 cells; stereological estimates of hFbs engraftment (n = 5) revealed an average of 11,701±3070 hFbs present 16 weeks following transplantation (Fig. 2F). These values represent a near 4-fold decrease of the initial cell population transplanted and suggest that hFbs did not survive well, nor did they proliferate.

During the sub-acute period a glial scar forms that includes growth inhibitory chondroitin-sulfate proteglycans (CSPGs) [37], [38], [39]. The properties of the glial scar and CSPGs contribute to an environment that is inhibitory to axonal regeneration and neurite outgrowth and has been suggested to prevent migration of transplanted cell populations [40], [41]. We have previously demonstrated glial scar formation and CSPG deposition (NG2 and Versican) in NOD-*scid* mice following SCI suggesting the NOD-*scid* SCI model presents an inhibitory proteoglycan environment to transplanted hCNS-SCns [30]. Accordingly, we investigated whether migration of hCNS-SCns and hFbs occurred following transplantation in the early chronic injury environment in which the glial scar and associated CSPGs are present. Migration of both hCNS-SCns and hFbs was assessed within the root dissected injury segments of the cord (T6–T12) that extends approximately 8 mm rostral and 5 mm caudal from the injury epicenter, including the spared tissue around the injury epicenter (vertical dashed line, Fig. 2G). The spared tissue area was defined as the intact region of cord surrounding the methyl green positive dense lesion core. We also quantified surviving cells within the lesion core, which both hCNS-SCns and hFbs tended to avoid. In this region, we counted only 5728±1317 hCNS-SCns and 303±169 hFbs, mostly localized to the lesion rim and rarely localized in the histological epicenter (Fig. 2G). hCNS-SCns migrated much further than hFbs, up to 8 mm rostral and 5 mm caudal compared to 2 mm rostral and 2 mm caudal for hFbs (Fig. 2G). At the distal rostral segments there were an average of 384 and 697 hCNS-SCns at 8 and 7 mm, respectively and at the distal caudal segments 1019 and 244 hCNS-SCns on average at 4 and 5 mm respectively. hCNS-SCns were concentrated in the spared tissue area and up to 4 mm rostral to the injury and 2 mm caudal. Similarly, hFbs were concentrated in the spared tissue area and up to 2 mm rostral to the injury, but hFbs did not migrate any further in the rostral direction and few cells were found in the region up to 2 mm caudal to the injury. The furthest rostral segments that hFbs migrated to contained an average of 3039 and 5769 hFbs at 5 and 4 mm, respectively and at the most caudal segment there were an average of 423 and 357 hFbs at 1 and 2 mm respectively. Both cell populations were more prominent rostral to the injury rather than caudal. Quantification revealed robust engraftment and migration of hCNS-SCns transplanted into an early chronic model of SCI.

### hCNS-SCns differentiate into all 3 CNS cell types

**Figure 3:**
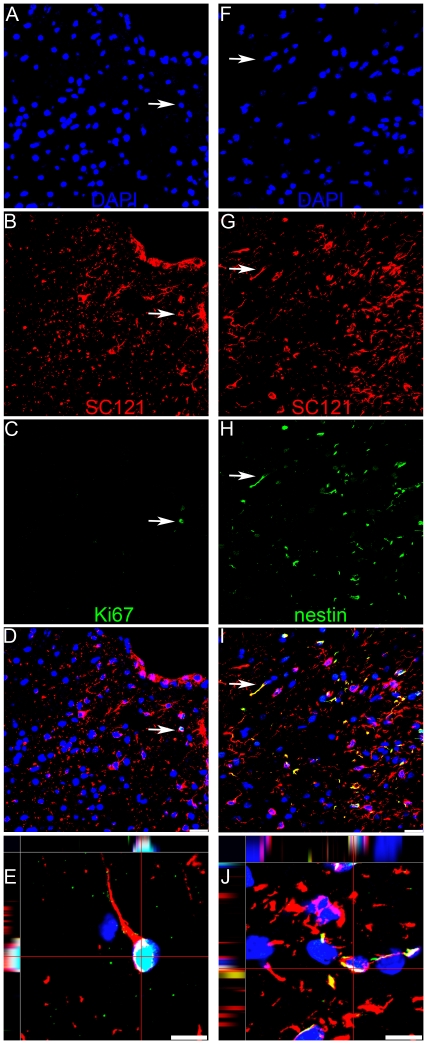
hCNS-SCns express Ki67 and nestin 16 weeks following transplantation. (**A–E**) Human cytoplasm-positive cells, (SC121), red (**B**) were rarely associated with the cell cycle marker Ki67, green (**C**), DAPI counterstain, blue (**A**). Arrows indicate a double-labeled cell. Merged confocal image, showing rare hCNS-SCns expression of Ki67 indicating low mitotic activity (**D**). Orthogonal view of confocal image showing co-localization of Ki67 and SC121 (**E**). (**F–J**) Some SC121 positive human cells, red (**G**) expressed the immature neural marker nestin, green (**H**) DAPI counterstain, blue (**F**). Arrows indicate a double-labeled cell. Merged confocal image reveals many hCNS-SCns maintain immature phenotypes and nestin expression 16 weeks after transplantation (**I**). Orthogonal view of confocal image revealing co-localization of nestin and SC121 (**J**). Scale bars  = 20 µm and 10 µm in the bottom row.

**Figure 4:**
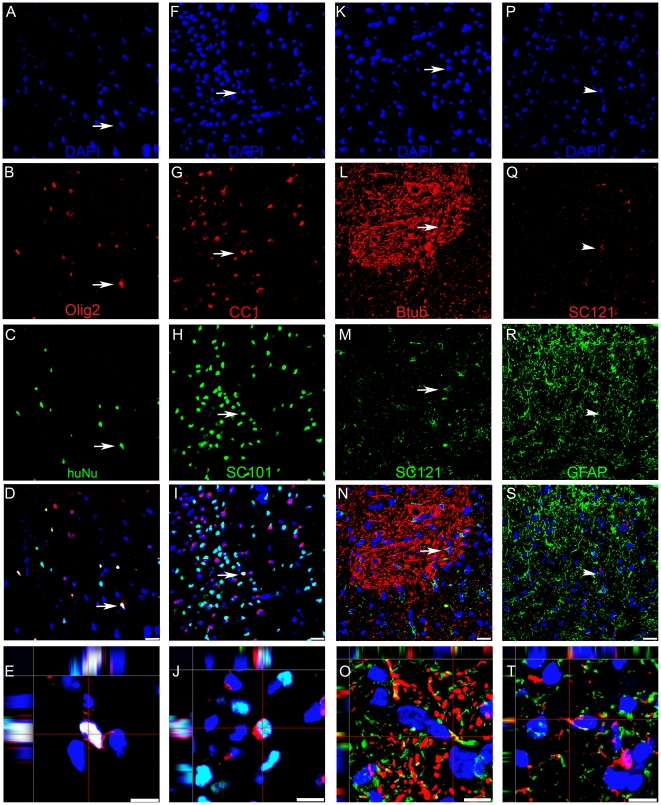
hCNS-SCns mostly differentiate into oligodendrocytes and neurons, and few astrocytes. (**A–E**) Several human nuclei positive cells, green (**C**), expressed Olig2 marker revealing immature oligodendrocytes, red (**B**), DAPI counterstain, blue (**A**). Arrows indicate a double-labeled cell. Merged confocal image, showing hCNS-SCns expression of Olig2 indicating differentiation to oligodendrocytes (**D**). Orthogonal view of confocal image showing co-localization of Olig2 and SC101 (**E**). (**F–J**) Some human nuclei-positive cells, SC101 green (**H**) also express the mature oligodendrocyte marker APC-CC1, red (**G**), DAPI counterstain, blue (**F**) Arrows indicate a double-labeled cell. Merged confocal image reveals some hCNS-SCns express APC-CC1 expression 16 weeks after transplantation. (**I**). Orthogonal view of confocal image revealing co-localization of APC-CC1 and human nuclei marker (**J**). (**K–O**) Human cytoplasm-positive cells SC121, green (**M**) also exhibit ß-tubulin III expression, red (**L**). DAPI counterstain, blue (**K**). Arrows indicate a double-labeled cell. Merged confocal image, revealing hCNS-SCns expression of ß-tubulin III (**N**). Orthogonal view of confocal image showing co-localization of ß-tubulin III and SC121 (**O**). (**P–T**) Few human cytoplasm cells SC121, red (**Q**) also expressed and the astrocyte marker GFAP, green (**R**). DAPI counterstain, blue (**P**) Arrowhead indicates a non-astrocytic human cell. Co-localization was rare indicating few hCNS-SCns exhibited astrocytic differentiation 16 weeks after transplantation (**R**). Orthogonal view of confocal image revealing co-localization of GFAP and SC121 (**S**). Scale bars  = 20 µm and 10 µm in the bottom row.

To investigate how the early chronic injured environment influenced the cell fate of engrafted hCNS-SCns, we assessed the fate and differentiation of hCNS-SCns by double immunofluorescence and confocal analysis in a subset of hCNS-SCns transplanted animals that were sectioned coronally. We investigated whether hCNS-SCns exhibited active cell division/proliferation by double labeling for SC121 and Ki67 and observed occasional co-localization (Fig. 3A–E). To determine whether any hCNS-SCns retained an immature phenotype we double labeled with SC121 and nestin and observed many co-localized cells (Fig. 3F–J). We next sought to determine whether engrafted human cells differentiated along oligodendroglial, neuronal, or astrocytic lineages. We investigated both immature and mature oligodendrocyte differentiation by double labeling for human nuclei and Olig2 to assess differentiation to immature oligodendrocytes (Fig. 4A–E) and APC-CC1 to examine mature oligodendrocytes (Fig. 4F–J). We observed hCNS-SCns double-labeled for both oligodendroglial markers. SC121-positive cells also co-localized with the neuronal marker β-tubulin III neurons suggesting hCNS-SCns differentiated into neurons (Fig. 4K–O). Few SC121 immunopositive cells co-localized with GFAP suggesting rare astrocytic differentiation (Fig. 4P–T).

**Figure 5:**
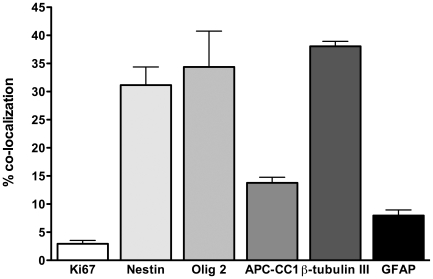
hCNS-SCns differentiation/fate quantification 16 weeks post-transplantation. Bar graph revealing quantification of hCNS-SCns that expressed the proliferative marker Ki67, the immature neural marker nestin, immature oligodendrocyte marker Olig2, the mature oligodendrocyte marker APC-CC1, the neuronal marker ß-tubulin III and the astrocytic marker GFAP expressed as percentages.

We investigated the percentage of human cells that exhibited evidence of active cell division/proliferation by double labeling for SC121 and Ki67. 2.9%±1.06 of SC121 positive human cells exhibited double labeling for Ki67 (Fig. 5), suggesting that there is limited proliferation of hCNS-SCns 16 weeks post-transplantation. Double labeling of SC121 and nestin revealed 31.1%±3.23 of SC121-positive cells retained nestin expression, suggesting that many human cells at 16 weeks post-transplantation remain immature (Fig. 5).

We next sought to determine the percentage of engrafted human cells that differentiated along oligodendroglial, neuronal, or astrocytic lineages. Quantification revealed 34.4%±6.38 exhibited differentiation along the more immature Olig2-positive oligodendrocyte lineage and 13.8%±1.0 of human cells differentiated into mature APC-CC1 positive oligodendrocytes (Fig. 5). Together, differentiation along an oligodendrocyte lineage comprised 40.78% of the human cells, which was the predominant fate of transplanted hCNS-SCns. 38.1%±0.85 of SC121-positive cells differentiated into β-tubulin III neurons, suggesting nearly as many hCNS-SCns differentiated into neurons as oligodendrocytes (Fig. 5). Few SC121 immunopositive cells differentiated into GFAP positive astrocytes, 8.0%±1.0 (Fig. 5).

Human myelinated host axons co-localize with contactin-associated protein

**Figure 6:**
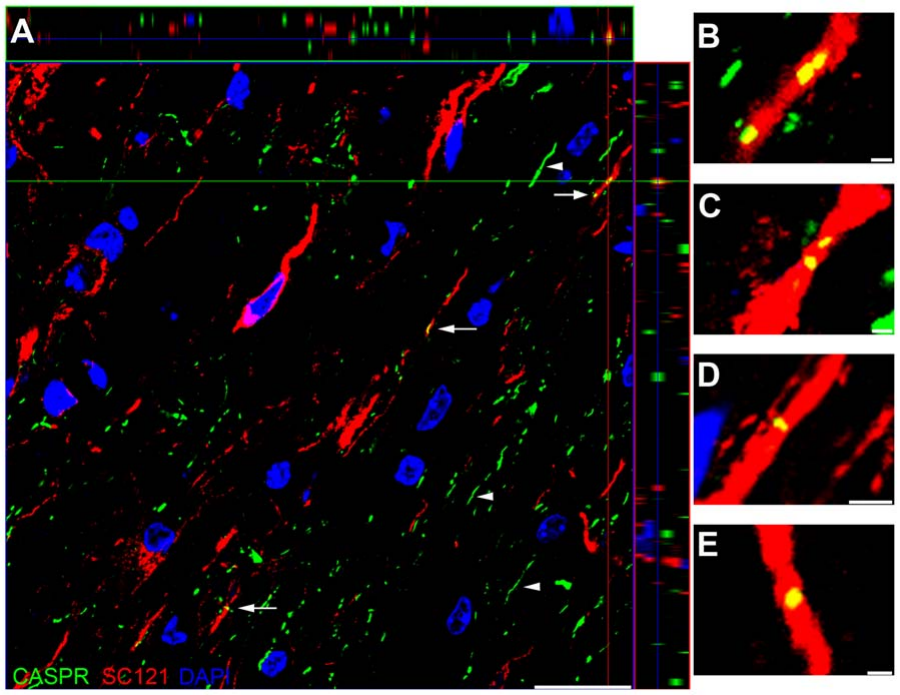
Human cytoplasm co-localizes with paranodal protein CASPR. (**A**) Orthogonal view of a confocal image of SC121 (red), CASPR (green) and DAPI counterstain (blue). The crosshair reveals co-localization of CASPR with SC121 and orthogonal projection. Arrows indicate additional SC121-positive axons exhibiting compact CASPR-positive paranodes. Arrowheads indicate diffusely distributed CASPR. (**B–E**) High-power images revealing examples of CASPR and SC121 co-localization. (**B**) High-power view of area in crosshair from (**A**). The two discrete CASPR-positive areas are ∼4 µm apart suggesting they are two paranodal regions of a single node. (**C**) High-power view of another co-localized axon revealing two discrete paranodal regions of a single node. (**D, E**) Additional high-power examples of SC121 co-localized with CASPR. Left scale bar  = 20 µm, right scale bars  = 1 µm.

hCNS-SCns in the white matter exhibited predominant oligodendrocyte differentiation while ß-tubulin III co-localization with SC121 was predominantly found within the gray matter and processes rarely extended to the white matter. We previously reported evidence that sub-acutely transplanted hCNS-SCns remyelinated host axons, we sought to determine whether we could visualize integration of human oligodendrocytes with the host within white matter [19]. CASPR is highly localized to the paranodal region of myelinated axons and can be used as an index of myelin integrity [42], [43], [44]; previous studies have demonstrated that CASPR has a compact paranodal restricted staining pattern in uninjured axons (Fig. 6A arrows) and this compact pattern becomes more diffusely distributed along the axon following SCI (Fig. 6A arrowheads) [43]. To visualize human myelinated host axons, we performed double immunofluorescence and confocal analysis for the human cytoplasmic marker SC121 (red) and CASPR (green) on parasagittal spinal cord sections 16 weeks following transplantation (Fig. 6). Staining revealed human myelinated host axons co-localized with compact CASPR in the white matter expressing paranodal proteins in the correct loci, suggesting hCNS-SCns integrated with the host.

### hCNS-SCns do not affect lesion volume, spared tissue volume, or glial scar area

**Figure 7:**
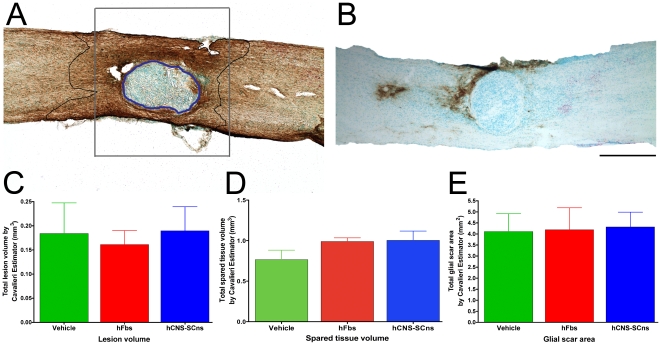
hCNS-SCns transplantation does not alter lesion volume, spared tissue volume, or glial scar area. (**A**) Representative spinal cord stained for GFAP to stereologically quantify lesion volume, indicated by the blue outline, spared tissue volume, quantified 500 µm rostrally and caudally from the lesion edges excluding the lesion, as depicted by the gray box, and the area of dense GFAP expression indicative of glial scarring excluding the lesion, outlined in black. (**B**) Staining of human GFAP (SC123), indicating rare astrocytic differentiation localized primarily near the injury site that did not exacerbate the glial scar. (**C**) Lesion volumes quantified using unbiased stereological probe Cavalieri Estimator show no significant difference for any of the three groups (p≥0.91 ANOVA). (**D**) Spared tissue volumes quantified using unbiased stereological probe Cavalieri Estimator show no significant difference for any of the three groups (p≥0.21 ANOVA). (**E**) Glial scar areas quantified using unbiased stereological probe Cavalieri Estimator show no significant difference for any of the three groups (p≥0.98 ANOVA). Scale bar  = 1000 µm.

While the presumptive strategy behind transplantation of stem cell populations for SCI has been cell replacement via integration as myelinating cells or new neurons, it is increasingly clear that transplanted cells can have a variety of effects on the host microenvironment including axonal regeneration and white matter sparing [9], [27], [45], [46], [47], [48], [49], [50]. To investigate the potential effects of early chronic transplantation on host parameters we assessed lesion volume, spared tissue volume, and glial scar area using methodology that we have previously employed that detected differences between complement knockout and wild type mice [28]. We have previously shown that hCNS-SCns transplantation 9 dpi did not alter host parameters of injury or repair [30]. We investigated host parameters of injury histologically by staining for the glial scar marker GFAP (Fig. 7A). Furthermore, using an antibody specific to human-GFAP (SC123) we investigated whether human astrocytes contributed to the host lesion or glial scar (Fig. 7B). The few human-GFAP cells observed were primarily localized near the injury epicenter (Fig. 7B). In the present study, lesion volume was quantified stereologically using the Cavalieri estimator probe in tissue stained for GFAP and counterstained with methyl green (Fig. 7A). Lesion volume was defined as the methyl green dense area devoid of GFAP staining (Fig. 7A), depicted with a blue outline. Vehicle treated animals had an average lesion volume of 0.18 mm^3^±0.06 (n = 5), hFbs treated animals 0.16 mm^3^±0.03 (n = 5), and hCNS-SCns treated animals 0.19 mm^3^±0.05 (n = 6). One-way ANOVA (p≥0.91) revealed no significant difference between any of the groups (Fig. 7C). We also quantified spared tissue, defined by 500 µm segments both rostral and caudal of the injury epicenter, excluding the lesion itself using Cavalieri estimator probe, (Fig. 7A gray box). Vehicle treated animals had an average spared tissue volume of 0.77 mm^3^±0.11 (n = 5), hFbs treated animals 0.99 mm^3^±0.05 (n = 5), and hCNS-SCns treated animals 1.00 mm^3^±0.11 (n = 6). There were no significant differences between any of the groups, one-way ANOVA (p≥0.21) (Fig. 7D). Glial scar area, defined as the dense GFAP staining surrounding the injury epicenter excluding the lesion core, depicted with a black outline, (Fig. 7A) was quantified stereologically using the Cavalieri estimator probe in all three groups to determine whether hCNS-SCns contributed to the glial scar (Fig. 7E). Vehicle treated animals had an average glial scar area of 4.11 mm^2^±0.80 (n = 5), hFbs treated animals 4.19 mm^2^±1.00 (n = 5), and hCNS-SCns treated animals 4.32 mm^2^±0.67 (n = 6). There were no significant differences in glial scar area between any of the groups (one-way ANOVA p≥0.98) suggesting that hCNS-SCns treatment neither exacerbated nor reduced the glial scar (Fig. 7E). Taken together, these data suggest that hCNS-SCns do not influence the lesion or spared tissue volumes, or glial scar area. We also assessed whether there were any correlations between lesion volume, spared tissue volume, or glial scar area with the number of engrafted hCNS-SCns. There were no significant correlations for any of these comparisons (data not shown).

## Discussion

### hCNS-SCns promote locomotor recovery

Our findings reveal hCNS-SCns transplanted into early chronic spinal cord injured NOD-*scid* mice survived, proliferated, and differentiated primarily into oligodendrocytes and neurons. This is the first study, to our knowledge, transplanting NSCs into early chronic SCI in which observed improved behavioral recovery has been detected. We assessed behavior via open-field locomotor testing (BMS) and CatWalk gait analysis. Our results showed improved recovery on BMS that was specifically enhanced in the range of coordination; hCNS-SCns treated mice exhibited a greater frequency of coordination compared to vehicle treated animals. Additionally, hCNS-SCns treated animals demonstrated improved swing speed compared to vehicle controls. hCNS-SCns transplantation did not result in detectable hind limb allodynia. Furthermore, transplanted hCNS-SCns had no measurable effect on lesion volume, spared tissue volume, or glial scar area. Taken together, these data suggest that stem cell transplantation can be successful outside the sub-acute time window and may be a potential therapeutic strategy for SCI at more chronic time points than commonly predicted.

### Human fibroblasts as a cellular control

In addition to a vehicle control we also utilized human fibroblasts as a cellular control in this study to assess whether the integration and differentiation of hCNS-SCns promotes improved recovery or whether any transplanted cell might affect behavioral recovery in either a detrimental or beneficial manner. Transplanted hFbs survived in all animals, but showed poor engraftment, with fewer than 25% of the initial transplanted population surviving 16 weeks later. Furthermore, hFbs did not migrate far, remaining near transplant sites. As we previously observed in a sub-acute transplant paradigm transplanting both hCNS-SCns and hFbs, animals receiving hFbs were intermediate in locomotor recovery between vehicle controls and hCNS-SCns transplanted animals, albeit non-significantly [19]. Taken together with the present study, the consistency of this finding suggests that hFbs do exert some effect on the injured spinal cord. hFbs could potentially improve locomotor recovery by secreting trophic factors that provide support to the injured cord. While it is clear there was limited survival and migration of hFbs, the data in this study cannot distinguish whether hFbs exhibited poor survival post-transplantation, or poor engraftment over time in the injured microenvironment. Perhaps greater engraftment of hFbs could also produce recovery of locomotor function following SCI, if it could be achieved. However, the lack of robust survival or proliferation of this cell population when transplanted into the parenchyma adjacent to the SCI epicenter, even in the absence of xenograft rejection suggests that this may not be possible.

### hCNS-SCns successfully engraft and migrate

Successful engraftment in CNS transplantation studies is very difficult to achieve, especially for xenografts. Currently, little is known about the dynamics of stem cell survival, proliferation, and migration in the SCI microenvironment because few studies have assessed these parameters. Furthermore, many studies fail to achieve adequate control of host immunorejection, making the assessment of these parameters very difficult. For stem cells to engraft in a therapeutically meaningful way, they need to survive both the initial transplantation and host-mediated rejection. The few studies that have quantified engraftment, in immunosuppressed xenograft or allograft models, as an endpoint report between 0.1% to 37% of the initial transplanted cells detected at time of sacrifice [6], [13], [36], [51], [52], [53], [54], [55], [56]. Additionally, few studies report the percentage of transplanted animals that are engrafted at the termination of the study, but under standard immunosuppression methods this is often as low as 50–60% in CNS injury models [57]. Because many studies fail to achieve successful engraftment this may explain why there has yet to be a successful report of NSCs improving recovery in a delayed transplant paradigm.

In contrast, constitutively immunodeficient animals yield better engraftment success, a study transplanting human NSCs into athymic nude rats quantified 275% more cells than initially transplanted [58]. Our studies have used NOD-*scid* mice, which are constitutively immunodeficient, lacking a normal T-cell, B-cell, and complement response. We have recently published a characterization of lesion volume, the innate immune response, and locomotor recovery in male and female NOD-*scid* mice in comparison with C57Bl/6 and BUB/BnJ mice [14], and report that NOD-*scid* mice do not exhibit differences in lesion characteristics and are within the range of strain variation for both macrophage and neutrophil responses. Therefore, despite the congenital deficiency in the adaptive immune response the cellular innate immune system is intact. While these mice also exhibit a partial complement deficiency (C5), this is true for most other mouse strains, including C57/Bl6, with the exception of BUB/BnJs [28]. Furthermore, NOD-*scid* mice have been used as a host for induced pluripotent cells in the CNS as an assay for tumor formation as well as NSC transplantation [16], [17]. Tumor formation is much more likely to occur in the absence of a rejection response therefore, NOD-*scid* mice allow for detection of tumor formation or abnormal growth of transplanted stem cell populations. However, one disadvantage of using NOD-*scid* mice is that due to the rate of spontaneous fatal thymoma formation the average lifespan of NOD-*scid* mice is roughly 8.5 months (34 weeks) limiting the length of studies that can be performed [59], [60]. Accordingly, NOD-*scid* mice provide an excellent experimental model to investigate the potential of transplanted human cell populations to engraft and promote histological and locomotor recovery following SCI without a xenograft rejection response [15]. As demonstrated in our sub-acute study, NOD-*scid* mice allowed for successful engraftment in 100% of transplanted animals [19]. Furthermore, upon sacrifice stereological quantification revealed 90% more hCNS-SCns than initially transplanted, demonstrating that this model permits proliferation [30]. The NOD-*scid* model has similar injury pathology to other mice strains but allows for successful engraftment and therefore an ability to assess the potential that human cell transplantation may have in experimental models of SCI.

In the present study, we assessed survival and migration of transplanted hCNS-SCns utilizing unbiased stereological quantification. All transplanted animals exhibited successful engraftment. Furthermore, we found 187% more hCNS-SCns than initially transplanted had successfully engrafted 16 weeks post-transplant, suggesting the transplanted cells are capable of limited proliferation. We also found long-distance migration from transplantation sites. Notably the engrafted cells avoid the lesion but occupy the spared tissue around the lesion. The cells migrated up to 8 mm rostrally and 5 mm in the caudal direction. Interestingly, more cells were found rostrally compared to caudally. This may be due to the rostral cord still receiving connections from the brain possibly contributing to a more trophic environment for transplanted cells. Conversely, a recent study suggested that regenerated sensory axons 6–8 months post-SCI remained unmyelinated rostral to injury but were myelinated caudally [61]. This may suggest that rostrally there is a need for oligodendrocyte replacement for remyelination of spontaneously regenerating or reorganizing sensory afferents. Therefore, the rostral migration and oligodendrocyte differentiation of transplanted hCNS-SCns may be filling this niche. hCNS-SCns transplanted into early chronic mouse SCI showed extensive engraftment, long-distance migration, and limited proliferation.

hCNS-SCns differentiate into oligodendrocytes and neurons

Approximately 31% of hCNS-SCns remained nestin positive suggesting that they remain undifferentiated, however of the cells that differentiated the majority differentiated along the oligodendrocyte lineage expressing the immature Olig2 marker or the mature APC-CC1 marker (41%) and nearly as many differentiated into ß-tubulin III-positive neurons (38%). The sum of all quantification markers was 128% suggesting there is overlap between some of the markers. Nestin, in particular has been found to co-localize with Olig2, β-tubulin III, and GFAP [62], [63]. Additionally, there could potentially be some overlap between Olig2 and APC-CC1 as cells are maturing along the oligodendrocyte lineage [64]. Interestingly, we observed increased neuronal differentiation in animals transplanted at 30 dpi compared to 9 dpi. hCNS-SCns may be responding to cues in the host microenvironment that are altered between these two time points resulting in the increased neuronal population. One of the variables changing over time following SCI is the continued influx of inflammatory cells. Our laboratory has recent data suggesting a delayed wave of infiltrating neutrophils, macrophages/microglia, and T-cells beginning about 21 dpi in Sprague-Dawley rat spinal cords and continuing through 6 months post-injury [65]. Post-mortem studies investigating human SCI tissue have also revealed the presence of macrophages chronically [66]. The cell types that are present in the spinal cord microenvironment at the time of cell transplantation and during differentiation may influence the fate of transplanted cells. Further studies investigating the factors that could contribute to increased neuronal differentiation when transplanting in the chronic SCI microenvironment are in progress. Furthermore, in contrast to many other studies that report predominant astrocytic differentiation after transplantation of NSCs into SCI models [21], [22], [23], [24], [25], [26] only about 8% of transplanted hCNS-SCns differentiated into astrocytes. Several studies from our lab and others have transplanted NSCs after SCI and also observed rare astrocytic differentiation [13], [19], [58]. Interestingly, these studies that observed rare astrocytic fate quantified at least 30% of transplanted cells successfully engrafted, potentially suggesting an immunological effect due to failure to prevent host immune rejection may promote astrocytic differentiation. However, there are many other variables that may promote astrocytic differentiation of transplanted NSCs after SCI including the source of transplanted NSCs, the culturing techniques, and cell preparation as well as potential differences between injury models. Further studies to elucidate the mechanisms resulting in astrocytic fate of transplanted NSCs are necessary and ongoing in our laboratory. Finally, a small percentage (2.9%) expressed Ki67 at the terminal time-point indicating that there is some limited continuing proliferation 16 weeks post-transplantation. However, no evidence of excessive proliferation, clusters of proliferating cells, or tumor formation was observed in any transplanted animals. NOD-*scid* mice have previously been used as a host for transplanted induced pluripotent cells and NSCs in the CNS as an assay for tumor formation [16], [17]. Tumor formation is much more likely to occur in the absence of a rejection response therefore, NOD-*scid* mice optimally allow for detection of tumor formation or abnormal growth of transplanted stem cell populations. Further studies investigating the kinetics of proliferation and migration at earlier time points following transplantation are in progress.

### hCNS-SCns integrate with the host

Because SC121-positive cells in the white matter predominantly differentiated into oligodendrocytes, co-localization of the human cytoplasmic marker SC121 with the paranodal protein CASPR within the white matter of the spinal cord is suggestive of host mouse axons remyelinated by hCNS-SCns that have differentiated into oligodendrocytes. ß-tubulin III co-localization with SC121 was predominantly found within the gray matter and processes rarely extended to the white matter. Despite SC121-positive axons rarely extending into the white matter the possibility that some of the observed co-localization with CASPR could represent myelination of these fibers by the host, cannot be excluded. Nonetheless, either case suggests that hCNS-SCns integrated with the host in this early chronic transplant paradigm. The mechanism of recovery is not known but integration of hCNS-SCns and remyelination of host axons is one possibility. Additional studies are necessary to further characterize the individual contributions of oligodendroglial and neuronal differentiation of hCNS-SCns in mediating locomotor recovery and establishing a mechanism of recovery. Critically, the CASPR staining pattern revealed both compact and diffuse CASPR, indicative of both normally myelinated and dysmyelinated axons 20 weeks after SCI in accordance with previous studies [43]. This suggests that dysmyelinated areas remain in the chronic SCI environment in NOD-*scid* mice, however the functional status of those axons cannot be determined [42]. It will be important to further characterize myelin pathology in the chronically injured spinal cord, however, the co-localization of SC121 and CASPR in this study suggest that it is possible to achieve hCNS-SCns-mediated remyelination of at least a subset of these fibers after transplantation in this early chronic SCI model which could contribute to recovery of function.

### hCNS-SCns do not affect lesion volume, spared tissue volume, or glial scar area

While the presumptive strategy behind transplantation of stem cell populations for SCI has been cell replacement via integration as myelinating cells or new neurons, it is increasingly clear that transplanted cells can have a variety of effects on the host microenvironment. In the present study, we investigated lesion volume, spared tissue volume, and glial scar area to determine whether chronically transplanted hCNS-SCns affected any of these host parameters. Because we observed improved recovery, we might expect the lesion size to be decreased [50], [67] **.** Alternatively, we might have expected transplanted cells to mediate recovery by increased sparing of white matter around the lesion [68]. We also investigated glial scar area to determine whether hCNS-SCns had reduced the size of the scar [49], [50]. Alternatively, hCNS-SCns that differentiated into astrocytes could have exacerbated the host glial scar. Stereological quantification of these parameters revealed no differences between hCNS-SCns and control groups. In a subsequent analysis of hCNS-SCns animals transplanted sub-acutely [19], lesion volume, tissue sparing, glial scarring, sprouting of host serotonergic fibers, and angiogenesis were investigated utilizing unbiased stereological quantification and no significant differences were detected between control and hCNS-SCns transplanted animals on any of these parameters [30]. Taken together with the sub-acute study and the effect of human cell ablation resulting in loss of locomotor recovery, these data support the hypothesis that hCNS-SCns transplantation after SCI mediates functional recovery by cellular integration with the host and not by overt modification of the host microenvironment.

### Chronic transplantation

Effective therapeutic NSC transplantation after SCI may only be possible if the transplanted cell population is capable of extensive migration, enabling cells to reach demyelinated and dysmyelinated axons and/or spared circuitry above and below the injury. Transplanted hCNS-SCns migrated multiple vertebral levels away from the transplantation site. Since there was significant oligodendroglial differentiation and we have previously demonstrated hCNS-SCns are capable of remyelination *in vivo*, one potential concern for chronic transplantation is whether demyelinated/dysmyelinated axons persist chronically [42]. Mammalian SCI models have exhibited evidence for chronic demyelination/dysmyelination in surviving and regenerating axons [43], [61], [69], [70], [71], suggesting remyelination as a viable therapeutic target in chronic SCI. Additionally, studies examining naturally occurring SCI in cats and dogs have found axons that are demyelinated up to 12 weeks post-injury [72]. In human chronic SCI tissue demyelinated and/or dysmyelinated fibers were observed [73], [74], [75]. Additionally, myelination during critical phases in the sub-acute and early chronic stages post-SCI could enhance axon sparing [42], [76], resulting in preservation of function. Remyelination may be a viable target for chronic transplantation, but may depend on timing in the acute to chronic injury continuum and requires further exploration. One could speculate that the preferential distribution of hCNS-SCns rostral to the epicenter and predominant oligodendrocyte fate could reflect a niche generated by the persistence of chronically demyelinated/dysmyelinated spared axons.

Collectively, following both sub-acute and early chronic transplantation we have shown that the predominant hCNS-SCns differentiation is oligodendroglial and survival of hCNS-SCns is required to sustain locomotor recovery, suggesting that oligodendrocyte integration with the host is likely a key mechanism of recovery [19]. However, the mechanism is not known and alternative pathways of hCNS-SCns-mediated repair must also be considered. We observed 26% of hCNS-SCns differentiated into neurons following sub-acute transplantation while 38% differentiated into neurons following early chronic transplantation suggesting a neuronal contribution [19]. Neuronal differentiation of transplanted hCNS-SCns could promote restoration of disrupted circuitry by formation of bridge or bypass connections. Neuronal replacement may be particularly useful in cervical SCI, where loss of motor neurons at the level of damage produce specific deficits, e.g. decreased triceps control. Neuronal differentiation could also provide trophic support to enhance neuroprotection and regeneration, or alter recruitment of endogenous progenitors that could contribute to repair processes. Additionally, NSCs can secrete a variety of neurotrophins in vitro and in vivo, including GDNF and NGF [77], [78]. Furthermore, human NSCs that exhibited neuronal differentiation have been shown to secrete GDNF following transplantation into traumatic brain injury [79]. Similarly, BDNF, GDNF, and NGF were present in higher amounts in human NSC transplanted animals that exhibited neuronal differentiation compared to controls following SCI [58]. Thus, despite predominant oligodendrocyte differentiation of hCNS-SCns, contributions of neuronal differentiation to improved locomotor recovery cannot be ruled out.

Previous studies have suggested SCI transplantation is more effective sub-acutely rather than chronically [12], [13], [54], [80]. However, our results suggest early chronic transplantation can still be effective for successful engraftment, differentiation to non-astrocytic lineages, and improvement of locomotor recovery. However, it is important to note that 30 dpi is a relatively “early” chronic time point and greater delays should be investigated. A relatively small proportion of the total SCI cases are new injuries that could benefit from potential acute therapies. If chronic therapies were developed, a much greater proportion of the SCI population would have the potential to benefit.

### Conclusion

The results of this study suggest that hCNS-SCns are capable of surviving and differentiating when transplanted in an early chronic injured microenvironment. The transplanted cells are not restricted to an astrocytic lineage and differentiate predominantly into oligodendrocytes and neurons. Furthermore, hCNS-SCns are capable of enhancing locomotor recovery. Overall these data suggest that hCNS-SCns transplantation may have potential as an intervention for SCI beyond sub-acute time points, which is of significant clinical relevance for the SCI population.
